# Epstein–Barr virus nuclear antigen-1 is useful as therapeutic efficacy marker in serum but not in saliva of nasopharyngeal cancer patients who underwent radiotherapy

**DOI:** 10.3332/ecancer.2021.1254

**Published:** 2021-06-21

**Authors:** Yurnadi H Midoen, Dwi A Suryandari, Luluk Yunaini, Raden Susworo, Elza I Auerkari, Hans-Joachim Freisleben

**Affiliations:** 1 Department of Medical Biology, Faculty of Medicine, Universitas Indonesia, Jalan Salemba Raya 6, Jakarta 10430, Indonesia; 2 Department of Radiotherapy, Faculty of Medicine, Universitas Indonesia and Cipto Mangunkusumo Hospital, Jalan Pangeran Diponegoro 71, Jakarta 10430, Indonesia; 3 Department of Oral Biology, Faculty of Dentistry, Universitas Indonesia, Jalan Salemba Raya 4, Jakarta 10430, Indonesia; 4 Medical Research Unit, Faculty of Medicine, Universitas Indonesia, Jalan Salemba Raya 4, Jakarta 10430, Indonesia; a https://orcid.org/0000-0003-1594-6475; b https://orcid.org/0000-0001-7604-8826

**Keywords:** NPC, EBV DNA, EBNA-1, qPCR, CT

## Abstract

**Introduction:**

Nasopharyngeal carcinoma (NPC) is a multifactorial disease with genetic, viral, environmental and lifestyle-related risk factors. Epstein–Barr virus (EBV) can promote the oncogenic transformation of an infected cell into malignant. EBV encodes many stimulating products including Epstein–Barr virus nuclear antigen-1 (EBNA-1) which plays a key role in the regulation of gene expression and replication of the genome in the latent period of infection. EBNA-1 in serum and tumour tissue of NPC patients correlates with NPC prognosis. Moreover, the presence of EBV DNA in serum samples from NPC patients’ blood circulation can be used as an early marker in the diagnosis of NPC.

**Objective:**

The objective of this study was to find effective methods for monitoring the progress of NPC patients undergoing radiotherapy and therapeutic efficacy by observing the changes in EBV DNA in serum and saliva.

**Methodology:**

The pre-experimental design compared blood and saliva taken from a pre-test and post-test group of NPC patients before and after radiation therapy. The concentration of EBV DNA was measured in the serum and saliva after amplification using quantitative polymerase chain reaction (qPCR) with compatible primers for the EBNA-1 gene. The data were statistically analysed by paired *T*-test.

**Results:**

Highly significant (*p* = 0.0001) increase in *cycle threshold* qPCR and decrease in the mean concentration of EBV DNA (p = 0.0001) were observed in serum samples, but no significant changes were observed in saliva.

**Conclusions:**

The results suggest that EBV DNA in serum can be used as the gold standard and a marker for monitoring the response to radiation therapy in NPC patients, whereas the examination of EBV DNA from saliva samples is not accurate and thus, not appropriate.

## Introduction

Nasopharyngeal carcinoma (NPC) is a multifactorial disease with primary genetic, racial, environmental, lifestyle-related risk factors including smoking, food intake and the uptake of carbon particles; the Epstein–Barr virus (EBV) is also closely related to the pathogenesis of NPC [1]. The NPC incidence shows endemic peaks in specific areas, particularly in southern China but also in Mediterranean Africa and some regions of the Middle East. [2].

Genetic factors are thought to play a role in the pathogenesis of NPC in high incidence populations of certain Asian (China, India, Vietnam, Thailand, Malaysia, Singapore and Indonesia) and African countries (Morocco, Algeria, Tunisia and Ghana [3, 4]). According to the International Agency for Research on Cancer, there were an estimated 129,079 new cases of NPC and 72,987 NPC-related deaths in 2018, worldwide [5]. Although the number of NPC cases in 2018 accounted for only 0.7% of all confirmed cancers, in some countries or regions NPC is at high risk. China has the highest incidence rate of NPCs worldwide. In 2018, there were 60,558 new NPC cases (47.7% of total global cases) and 31,413 NPC-related deaths in China [1, 5].

In Indonesia, which has an ethnically diverse population of 225 million people, NPC is prevalent among different indigenous peoples and presents a significant socio-economic problem, with an estimated overall incidence of 6.2 per 100,000 or around 12,000 new cases per year [6], thus being the most prevalent malignant tumour of the head and neck region and the fourth-most common malignant cancer after cervical, breast and skin cancers [7]. Its incidence is considered to be quite evenly spread across the provinces. In the ENT Department of Cipto Mangunkusumo Hospital (Rumah Sakit Cipto Mangunkusumo, RSCM), the national Indonesian hospital in Jakarta, there are more than 100 cases of NPC per year, 60 cases per year in Hasan Sadikin Hospital Bandung, 25 cases in Makassar, 25 cases in Palembang, 15 cases in Denpasar, 11 cases per year in the Padang and Bukit Tinggi region, similar to Medan, Semarang, Surabaya and other cities.

Aetiological factors such as EBV infection and carcinogenic substances contribute to NPC tumorigenesis in complex interactions [2, 8]. This was demonstrated by multiple gene polymorphisms with different genetic characteristics carried by ethnic groups in high-risk areas associated with varying degrees of NPC risk as well as processing and presentation of EBV antigens. Extracts of Cantonese salted fish as well as herbal medicines used by Chinese have been shown to increase the activation and proliferation of EBV leading to NPC [9].

EBV cannot be found in normal nasopharyngeal epithelial cells, but the genome of EBV is present in the tumour cells of (WHO-II) NPC [8]. For example, EBV genes were detected in the majority of tissue samples from Sudanese patients with NPC [10]. Ayee *et al* [11] analysed the association of EBV infection in patients diagnosed with NPC in Ghana. The EBV load was significantly higher in NPC patients than in controls; thus, its assessment can be used as a biological marker for the diagnosis of NPC.

Stable EBV infection and latent EBV gene expression can promote the oncogenic transformation of pre-invasive nasopharyngeal epithelial cells into malignant cancer cells through several pathways [12, 13]. In the circular EBV episome with 171.823 base pairs (bp), the double helix DNA genome represents approximately 85% of the activated genes [14]. Furthermore, EBV encodes many stimulating products, such as Epstein–Barr virus nuclear antigen-1 (EBNA-1), EBNA-2, EBNA-3A, EBNA-3B, EBNA-3C, Epstein-Barr virus nuclear antigen leader protein (EBNA-LP), latent membrane protein-1 (LMP-1), EBV-encoded RNA-1 (EBER-1), complementary strand transcript (CST) or Bam A rightward transcript (BART) [15, 16]. All of them can have interaction or homology with body proteins, such as antiapoptotic proteins, cytokines and signal transducers that can promote EBV infection, immortalization and cell transformation [13, 15]. Among these candidates, EBNA-1 gene was found to be the most frequent in Vietnamese NPC patients [17, 18].

According to Lu *et al* [16], EBNA-1 plays a key role in the regulation of gene expression and replication of the genome in the latent period of infection. EBNA-1 is a DNA-binding protein with strong activity to bind RNA and is important in keeping the EBV genome in B-cells as a circular DNA episome. EBNA-1 in serum and tumour tissue of NPC patients correlates with NPC. Moreover, the presence of EBV DNA in the serum from NPC indicates the presence of viral DNA in the circulation which in turn can be used as an early marker in the diagnosis of NPC [19].

Using nested polymerase chain reaction (PCR), Banko *et al* [20] found that subvariant-5 threonine polymorphism (P-Thr-sv-5) can show the variability of EBNA1 specific carcinoma in tissue and plasma of patients with undifferentiated NPC, monocytosis syndrome and kidney transplantation. The authors suggested that the identification of this subvariant could be used as a virus screening marker for the identification of NPC. However, further *in vitro* studies are needed to ascertain whether additional amino acid substitution of this subvariant will affect the function of EBNA1 in regulating the replication and transcription of the EBV gene and/or alter the effect of EBNA1 on transcription of other genes in the host cell [21].

Serological NPC diagnosis can be established by detecting immunoglobulin A (IgA) towards EBV lytic gene products like the early antigen and viral capsid antigen (VCA) that increase in the serum [3]. Serological tests of EBV are associated with the discovery of antibodies (IgA-VCA) used for NPC screening and as tumour markers to aid the early diagnosis of NPC patients [13, 22]. Furthermore, quantitative analysis showed that the presence of EBV DNA can be used as a sensitive indicator to detect and confirm NPC [23]. The study found that the EBV DNA level in plasma of NPC patients is a more sensitive marker than IgA-VCA, and that the levels of EBV DNA measured in plasma can be used for early diagnosis, monitoring local recurrence, distant metastasis and accurate prognosis of NPC patients [24].

EBV DNA can be detected by amplification of EBNA-1 gene using quantitative PCR (qPCR) from tumour tissue and body fluids of NPC patients. Therapy can be effective in WHO-II type NPC patients if it decreases EBV DNA in the circulation and eliminates tumour cells, thus minimising the possibility of reactivation of the virus that can induce again the growth of a new tumour (recurrence). Nevertheless, the aspects of molecular genetics in NPC are not fully evaluated, such as the presence of EBV DNA in serum and saliva of NPC patients undergoing therapy. In particular, no experiments on Indonesian NPC patients have been reported in this context [25].

This study aims to find effective methods to monitor the progress and therapeutic efficacy on NPC by observing the changes in the presence of EBV DNA in the serum and saliva of NPC patients undergoing radiotherapy.

## Materials and methods

### Subjects

This study was conducted in the Department of Radiotherapy of RSCM and Department of Medical Biology, Faculty of Medicine, Universitas Indonesia (FMUI) from April to November 2012. The subjects were patients with advanced stage of NPC (stages III and IV), confirmed through clinical and histopathological methods. All patients were treated by radiotherapy and had given written informed consent to participate in the study. Diagnosis of NPC was established due to the histopathological examination of tissue biopsies by the Department of Anatomical Pathology FMUI/RSCM, Jakarta. Staging of NPC according to the American Joint Committee on Cancer or *L’Union Internationale Contre le Cancer* (*UICC*) was classified by EIA and an anatomical pathologist from FMUI/RSCM based on WHO criteria for type 1, 2 and 3. The demographic data, patient histories and clinical information were obtained using a questionnaire and medical records.

This study is considered pre-experimental using one-group pre-test and post-test design. Peripheral blood samples (3 mL) and morning saliva samples were taken from NPC patients, before the start and after completion of radiation therapy. Patients’ saliva was taken into 5–10 mL sample containers and stored in a freezer at −20°C until analysed.

Radiation therapy was delivered by a Linear Accelerator, 33–35 times within 6 to 7 weeks, depending on the stage of the tumour. Only patients who completed the treatment according to the planned protocol were enrolled in this study. If the patient did not fully complete the therapy (33–35 times within 6 to 7 weeks), he/she was not included in this study.

### Isolation of viral DNA

Peripheral blood (3 mL) was collected from NPC patients using vacutainers with ethylenediaminetetraacetic acid (EDTA) anticoagulant. The blood samples were centrifuged to separate serum and blood cells. EBV DNA was isolated as follows [26]: 100 mL serum/saliva was inserted into a 1.5 mL Eppendorf tube followed by the addition of 300 mL Tris-EDTA (TE), 50 mL sodium dodecyl sulfate (SDS) 10% and 1 mL proteinase-K. The tube was vortexed for about 1 minute and incubated at 65°C for 1 hour. Then, 200 mL phenol and 200 mL of chloroform iso-amyl-alcohol 24:1 were added; the solution was vortexed for about 3 minutes and centrifuged at 10,000 rpm for about 10 minutes at 20^°^C to separate the aqueous layer which was transferred into a new Eppendorf tube.

In the next step, 30–40 mL of sodium acetate, pH 4.2, and 1 mL of cold absolute ethanol, was added to precipitate DNA by gently turning the Eppendorf tube several times and then incubating overnight at −20°C. The tube was then centrifuged at 12,000 rpm for 30 minutes at 20°C until a white substance was visible at the bottom of the tube. After discarding the supernatant, cold sterile 70% ethanol was added to wash DNA,and the solution was centrifuged at 10,000 rpm for 10 minutes at 20°C. After carefully removing and discarding the supernatant, the remaining DNA was dried for 30 minutes. The dried DNA pellet was then rehydrated with 20 mL TE and stored at −20°C for later analysis.

### Primer design of EBNA-1 gene (EBV DNA)

The EBNA-1 gene was chosen to detect EBV DNA because it is conserved and expressed in all EBV genomes, both in latent and lytic phases. After obtaining the sequence of EBNA-1 gene, two sets of EBNA-1 primers were designed on the website of Primer3 output [27, 28]. This resulted in an outer left primer 5ʹCAGAAAGGCCTCGAGCTGTʹ3, outer right primer 5ʹCCAGAGGATGCCCTGAGACTʹ3, inner left primer 5ʹGCACCTCCTTCTGTCTGAGCʹ3 and inner right primer 5ʹACTCTCTGGGCTGCAGAATCʹ3.

### Amplification of EBV DNA with nested-PCR (2-step PCR)

EBV DNA amplification was applied using two sets of primers and the method of nested-PCR. Each 50 mL reaction mixture for amplification contained 10 L DNA template, 200 mM deoxyribonucleotide triphosphate (dNTP, combination of deoxythymidine triphosphate (dTTP), deoxycytidine triphosphate (dCTP), deoxyguanosine triphosphate (dGTP), deoxyadenosine triphosphate (dATP)), 1.25 units of Taq DNA polymerase, buffer solution of 10 mM Tris-HCl at pH 9, 50 mM KCl, 0.1% Triton X-100, 1.5 mM MgCl_2_ and ddH_2_O. For the first step of PCR reaction, 10 pmol EBNA-1 outer left and outer right primers were added. As negative control, a combination of all PCR reagents was used with 10 mL ddH_2_O added.

Amplification used several cycles of denaturation, annealing primer and extension of DNA in the PCR machine. Early denaturation (pre-PCR) was done at 95°C for 9 minutes, followed by a period of PCR with 35 cycles consisting of denaturation at 94°C for 30 seconds, annealing at 65°C for 60 seconds and extension at 72^0^C for 60 seconds. After several periods, PCR was ended with final extension (post-PCR) at 72^0^C for 10 minutes.

In the second PCR step, 10 mL DNA sample from the first PCR step was used as the template for amplification in a 50 µL reaction mixture containing 200 mM dNTP (combination of dTTP, dCTP, dGTP, dATP), 1.25 units of Taq DNA polymerase, buffer solution of 10 mM Tris-HCl at pH 9, 50 mM KCl, 0.1% Triton X-100, 1.5 mM MgCl_2_ and ddH_2_O. The second PCR step used the same EBNA-1 primers, negative control and amplification method as described in the previous step.

### Detection of EBV DNA

Amplicons of DNA from patient samples (serum or saliva) before therapy were checked by separating the DNA fragments with horizontal electrophoresis using 2% agarose gel with 1 mg/mL ethidium bromide in the buffer of Tris-acetate-EDTA (TAE) 1x. Into each well, a suspension was added consisting of 10 mL EBV DNA and 3 mL loading buffer (0.25% bromophenol blue, xylene cyanole, 4% sucrose) and separated by electrophoresis at 90 V for up to 60 minutes. As a marker, a DNA ladder of 100 bp was used. The DNA fragments separated by electrophoresis were detected using a UV illuminator and a 125 bp band of EBV DNA was considered positive result. The DNA bands were recorded on Polaroid film.

### Measurement of the EBV DNA concentration by qPCR

To measure EBV DNA concentrations, qPCR was applied with two primers that had been used to evaluate EBNA-1 and produce the DNA amplicon of 125 bp: left side (forward) primer of 5ʹGCACCTCCTTCTGTCTGAGCʹ3 and right side (reverse) primer of 5ʹACTCTCTGGGCTGCAGAATCʹ3. The qPCR mix was composed of real-time PCR (RT-PCR) master mix kit Qiagen (containing HotStar Taq plus DNA polymerase, rotor gene SYBR Green RT-PCR buffer, dNTP mix), 0.2 μM primer and 50 ng cDNA template at a total volume of 25 µL. Amplification was performed on a RT-PCR machine (Rotor-Gene Q, Qiagen) with a total of 40 PCR cycles, initial denaturation at 95°C for 5 minutes, denaturation at 90°C for 5 seconds, annealing and elongation at 60°C for 10 seconds. The standard curve was made by stratified dilution of the concentration of EBNA-1 EBV DNA samples, measured with NanoDrop as positive control (NanoVue GE) as follows: 10^-1^, 10^-2^ and 10^-3^.

### Statistical analysis

The analysis of the qPCR results ruled out the presence of EBV DNA. The qPCR results were calculated and data grouped before statistical analysis (data are nominal). The EBV DNA concentration data were obtained before and after therapy and compared with paired *T*-test.

## Results

### Amplification of EBV DNA with primers comprising EBNA-1 gene

**Figure 1.**
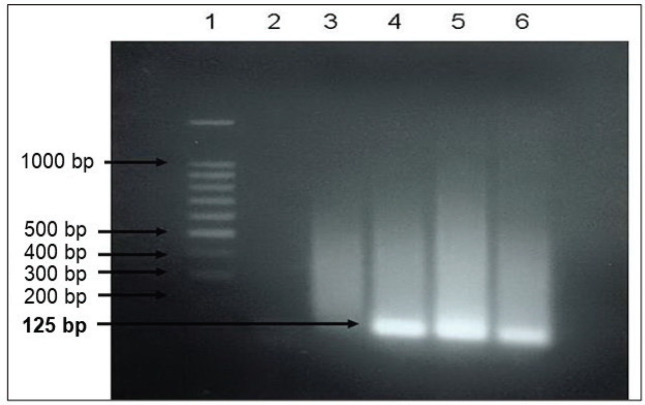
Results of nested PCR after electrophoresis. 1 = marker, 100 bp DNA ladder, 2 = negative control; 3 = serum sample negative for EBNA-1; 4 to 6 = serum samples positive of EBNA-1.

Amplification of EBV DNA gene (EBNA-1) with nested PCR resulted in a DNA band of 125 bp (Figure 1), suggesting that the primer design was correct and the band of 125 bp indicated real EBV DNA.

### Determination of EBV DNA in serum by qPCR

**Figure 2.**
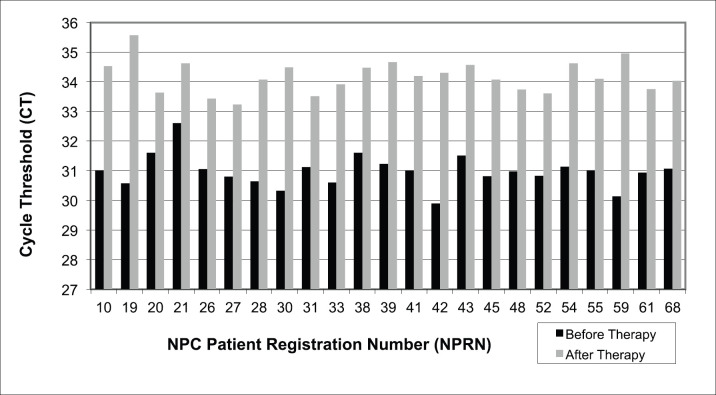
EBV DNA determination by qPCR in serum of NPC patients before and after therapy. CT = cycle threshold, lower CT indicates higher amount amplification of the target nucleic acid in the sample and vice versa for higher CT. Paired *T*-test, *p* = 0.0001.

EBV DNA was determined by qPCR cycle threshold (CT) value in 23 serum samples from NPC patients. All samples showed a significant increase in qPCR CT value after therapy as compared to samples taken from the same patient before therapy (Figure 2).

Concerning the existence of EBV DNA, the value of qPCR CT and the concentration of its amplification results from the serum of NPC patients before and after therapy are counter-current or inverse, i.e. all serum samples of NPC patients show an increase in CT values and a decrease in the concentrations of EBV DNA amplification (Figure 2 and Table 1).

The average CT value increased from 30.98 before to 34.18 after therapy. Paired *T*-test between these groups obtained highly significant difference at *p* = 0.0001. This increase in qPCR CT values would subsequently have an impact on the reduction of EBV DNA amplification during qPCR. EBV DNA concentration in qPCR amplification decreased almost 180-fold from an average of 0.02324 ± 0.0056 ng/mL before to 0.00013 ± 0.00008 ng/mL after therapy (Table 1). Paired *T*-test resulted in highly significant difference at *p* = 0.0001. Thus, in serum, EBV DNA concentration from qPCR amplification after therapy is significantly lower than before.

### Detecting EBV DNA in saliva by qPCR

**Figure 3.**
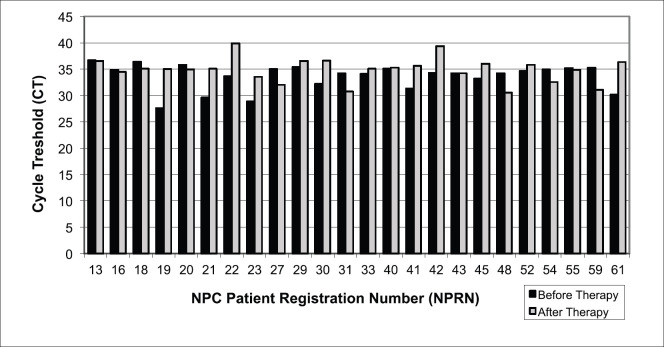
EBV DNA detection by qPCR in NPC patient saliva before and after undergoing therapy. CT = cycle threshold, lower CT indicates higher amount amplification of the target nucleic acid in the sample and vice versa for higher CT. Paired T-test, *p* = 0.835.

EBV DNA was determined by qPCR (CT) value in 24 saliva samples from NPC patients. The existence of EBV DNA from the aspect of CT values and the EBV DNA concentration resulting from its amplification in the saliva of NPC patients was not consistent (Figure 3 and Table 2). Some samples indicated an increase in CT values after therapy, some indicated a decrease and some others seemed stable (Figure 3). As a result of inconsistently varying differences in CT values before and after therapy, EBV DNA concentration resulting from amplification in qPCR of each NPC patient showed similar inconsistency with decrease, stability and increase (Figure 3 and Table 2). However, there was also an inverse relationship between CT values and EBV DNA concentration by qPCR.

The average CT value of qPCR increased from 33.61 before to 34.90 after therapy. The result of *T*-test when comparing CT values from saliva samples before and after therapy did not show significant differences (*p* = 0.835). The insignificant increase in CT values of qPCR from saliva DNA EBV was not expected to subsequently have a significant impact on the course (decline or increase) of the EBV DNA amplification during qPCR (Table 2).

**Figure 4.**
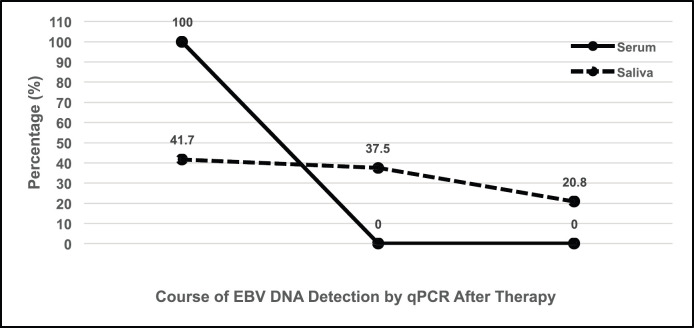
Schematic tendency of the detection of EBV DNA by qPCR in the serum and saliva of NPC patients after therapy; whereas in 100% of serum samples, the concentration drops by two magnitudes towards zero, in 41.7% of saliva samples the concentration remains about the same, in 37.5% it decreases, and in 20.8% the concentration even increases.

The average concentration of EBV DNA before therapy was 0.00031 ng/μL and decreased to 0.00018 ng/μL after therapy. The EBV DNA concentrations after amplification by qPCR decreased in 37.5%, remained similar in 41.7% and increased in 20.8% of all 24 cases (Figure 4). As expected, the results of paired *T-*test on the EBV DNA concentration after amplification by qPCR between the groups before and after therapy did not reveal significant differences at *p* = 0.2925. Therefore, saliva samples of NPC patients are not considered useful to produce clear and significant results.

## Discussion

The EBNA-1 gene was chosen as a marker to detect EBV DNA, because it is well conserved, not polymorphic and active in both lytic and latent phases. Amplification of EBV EBNA-1 gene by nested-PCR produced a DNA band of 125 bp demonstrating that EBV DNA can be detected from both saliva and serum of NPC patients.

Although EBV DNA purity was below the value of the raw purity, nested-PCR produced the expected results with the design of specific primers and the right method of inhibiting EBV DNA amplification.

Our results differ from those of a previous study assessing the presence of EBV DNA semi-quantitatively through the thickness of EBV DNA bands from nested-PCR [29]. In particular, the results of the present work refute the results of the previous study which claimed that for the presence of EBV DNA, testing saliva was better than serum. We conclude that EBV DNA decreases faster in serum than in saliva, indicating that serum tests assessing the presence of EBV DNA in NPC patients are more informative on the therapeutic effectiveness.

This is consistent with previous work on NPC patients with no EBV DNA detected in plasma but showing complete tumour response [18]. The presence of EBV DNA in NPC patients before and after therapy is also associated with tumour size. Free EBV DNA is usually detected in the plasma of all patients with NPC.

Two months after therapy, the tumour mass has usually disappeared from the patient’s body and the NPC healing process will continue. However, if the healing process is not completed within 2 months after therapy, remnant tumour tissue may still be present, which needs to be confirmed by detection of EBV DNA. If EBV DNA is found at high levels after recovery, the virus is still replicated in the remnant NPC tissue and released into the circulation [30].

The release EBV DNA into plasma of NPC patients may be a marker of cell death [31]. Apoptosis in tumour tissues appears to correlate with the presence of EBV DNA in the serum of NPC patients [32]. Persistence of EBV DNA in the serum is expected to result from the process of metastasis.

Using RT-PCR, EBV DNA has been detected in the plasma of about 96% of the NPC patients, with 21,058 copies/mL, and in 7% of control patients (0 copy/mL) [23]. In preliminary measurements of EBV DNA in patients with advanced stage of NPC (stages III and IV), higher levels of EBV DNA were found as compared to the early stage (stages I and II). After 1 month undergoing radiotherapy, EBV DNA in the plasma was no longer detected in 47% of the patients but remained at considerably high levels in 53% of the patients. In the clinical trials, seven patients had complete tumour regression, but out of eight patients who showed DNA EBV in plasma after therapy, six had persistent NPC because of incomplete tumour regression or even developed metastases [23].

These results suggest that measuring plasma EBV DNA can be applied to indicate the risk of NPC persistence after radiotherapy and to identify patients who will require more aggressive therapy like combined chemo-and radiotherapy.

Another study using RT-PCR of plasma from former NPC patients before and after therapy showed that in NPC patients who had not undergone radiotherapy, EBV DNA concentrations were clearly higher than in healthy controls and in NPC patients who had undergone radiotherapy [33].

The clinical significance of measuring the EBV DNA concentration in the plasma of advanced stage NPC patients (stages III and IV, with and without metastases) with RT-PCR was considered 10 weeks after radio-chemotherapy [34]. In cases that had not undergone therapy,

EBV DNA in the plasma was detected in 94 out of 99 patients (94.9%), but in none of the 40 control patients. After therapy, EBV DNA was detected on the average level of 681 copies/mL in 25 patients with stage III, of 1,703 copies/mL in 74 patients with stage IV and of 291,940 copies/mL in 19 patients with metastases (*p* < 0.001). It is concluded that the quantification of EBV DNA in blood plasma can be used to monitor NPC patients and predict the need for further therapy or interventions.

There is also potential for even less invasive monitoring of NPC, as a positive correlation of EBV DNA appears to exist in plasma and in urine [35].

Gihbid *et al* [36] evaluated the correlation between pre-treatment plasma EBV DNA load and conventional prognostic factors in Moroccan NPC patients. Measuring the pre-treatment EBV plasma load, 90.9% of the patients had detectable EBV DNA, with a mean plasma viral load of 7,710 IU/mL. The correlation between pre-treatment EBV DNA load and conventional prognostic factors showed a significant association with patients’ age, tumour classification, lymph node status, metastatic status and overall cancer stage (*p* = 0.01). The results of this study clearly demonstrated a high association between pre-treatment EBV DNA load, important classical prognostic factors (T, N, M and disease stage) of NPC and suggested that pre-treatment EBV DNA load can be a useful prognostic biomarker and improve the treatment of NPC [36].

Liu *et al* [37] conducted a meta-analysis study predicting prognosis and therapeutic outcomes in NPC patients by evaluating the number of copies of plasma EBV DNA at pre-treatment as an outcome marker for survival in NPC. A total of 16 studies with 7,698 patients found that high levels of EBV DNA indicated a poor prognosis and reduced long-term survival in patients with newly diagnosed NPC. Therefore, EBV DNA levels are highly prognostic for survival in patients with NPC [37]. Furthermore, Li *et al* [38] reported that long-term monitoring of dynamic changes in plasma EBV DNA may improve the prognostic prediction of NPC. The dynamic changes in plasma EBV DNA before and after therapy may predict long-term survival and provide accurate risk stratification for NPC.

Huang *et al* [39] examined the prognostic value of EBV DNA after induction chemotherapy (ICT) and concurrent chemoradiation (CCRT) as predictors of locoregional NPC. The authors found that the amount of EBV DNA in the patients’ plasma after undergoing ICT showed good predictive results and could be used as an early predictor of NPC. This parameter could monitor the development of locoregional NPC and could be used to guide therapy modification strategies.

Lai *et al* [40] studied the efficacy of ICT followed by concurrent chemoradiotherapy (CCRT) against locoregional advanced NPC (LA-NPC). They considered pre-treatment plasma EBV DNA levels to be a promising effective marker for ICT use in LA-NPC patients. Addition of ICT could increase overall survival and progression free survival in patients with an EBV DNA load of ≥ 4,000 copies/mL, but no efficacy was found in patients with an EBV DNA load of < 4,000 copies/mL. In addition to EBV DNA levels, ICT can also increase distant metastasis-free survival and has no effect on locoregional relapse-free survival [40].

Li *et al* [41] conducted a study to determine plasma EBV DNA levels pre-treatment and post-treatment (3 months-EBV) as an important biomarker in the prognosis of NPC. It was found that integrating EBV DNA data pre-treatment and post-treatment (3 months-EBV) could predict an accurate and prognostic risk level for NPC [41].

Although the combination of radiotherapy with chemotherapy has become the accepted standard in locally advanced NPC, during the time when our study was conducted, there were many patients in Indonesia treated by radiotherapy alone. Anyways, this fact may be a limitation of our study and follow-up should be considered with combination therapies.

## Conclusion

In all 23 serum samples from NPC patients undergoing therapy, a decrease in EBV DNA concentrations after qPCR amplification was detected by 100%. In contrast, out of 24 saliva samples, EBV DNA concentration after qPCR amplification decreased in nine NPC patient samples (37.5%), remained similar in ten (41.7%) and even increased in five patient samples (20.8%) after therapy.

In serum samples, but not in saliva samples, the CT qPCR of EBV DNA consistently and highly significantly increased and the average EBV DNA concentration significantly decreased after radiotherapy. The detected level of EBV DNA in serum can indicate the degree of success in NPC patients after therapy.

Further and larger-scale studies are recommended on the detection of EBV DNA in the serum of normal individuals to determine the risk of developing NPC. It is also necessary to assess the status of EBV DNA in NPC patients for several years after therapy to evaluate of the role of EBV DNA in the recurrence of NPC.

## Authors’ contributions

YHM carried out molecular-genetic and PCR experiments, statistical analyses and wrote up the draft of the manuscript; LY cooperated with YHM, did DNA isolation, PCR experiments, designed the graphics and compiled the tables; RS carried out the radiotherapy, EIA conducted the histopathology, NPC classification including questionnaires and wrote up this part of the manuscript. DAS designed, organised and supervised the study, provided funding and helped with the Discussion. HJF participated in drafting the manuscript, improved the presentation of the Results and the Discussion and revised the final version of the manuscript.

## Conflicts of interest

The authors have no conflicts of interest to declare.

## Figures and Tables

**Table 1.** The calculation of EBV DNA concentration (EBNA-1) by qPCR in serum of NPC patients before and after undergoing therapy.

NPRN	Calculation of concentration of EBV DNA (ng/mL)	
	Before therapy	After therapy
10	0.0241	0.000285
19	0.0270	0.000211
20	0.0208	0.000107
21	0.0161	0.000278
26	0.0239	0.000117
27	0.0255	0.000127
28	0.0265	0,000325
30	0.0288	0.000288
31	0.0235	0.000113
33	0.0268	0.000095
38	0.0209	0.000075
39	0.0229	0.000069
41	0.0242	0.000084
42	0.0011	0.000081
43	0.0213	0.000072
45	0.0253	0.000089
48	0.0244	0.000102
52	0.0253	0.000108
54	0.0234	0.000071
55	0.0241	0.000088
59	0.0301	0.000061
61	0.0246	0.000102
68	0.0238	0.000090
Average	0.02324	0.00013
Standard deviation (SD)	0.0056	0.00008
Standard error of mean (SEM)	0.0012	0.0001

Note: NPRN (NPC patient registration number)

**Table 2.** The calculation of EBV DNA concentration (EBNA-1) by qPCR in saliva of NPC patients before and after undergoing therapy.

NPRN	Calculation of concentration of EBV DNA (ng/mL)	
	Before therapy	After therapy
13	0.000045	0.000026
16	0.000097	0.000090
18	0.000050	0.000062
19	0.001780	0.000065
20	0.000064	0.000067
21	0.001190	0.000063
22	0.000150	0.000003
23	0.001060	0.000158
27	0.000088	0.000592
29	0.000109	0.000025
30	0.000275	0.000025
31	0.000121	0.000877
33	0.000130	0.000061
40	0.000085	0.000054
41	0.000388	0.000045
42	0.000120	0.000005
43	0.000122	0.000104
45	0.000183	0.000035
48	0.000121	0.000905
52	0.000105	0.000039
54	0.000090	0.000293
55	0.000081	0.000069
59	0.000080	0.000774
61	0.001010	0.000029
Average	**0.00031**	**0.00018**
SD	**0.00045**	**0.00028**
SEM	**0.00009**	**0.00006**

Note: NPRN (NPC patient registration number)
